# An Assessment of Risk Factors of Delayed Speech and Language in Children: A Cross-Sectional Study

**DOI:** 10.7759/cureus.29623

**Published:** 2022-09-26

**Authors:** Anish Kumar, Maryam Zubair, Azouba Gulraiz, Sruti Kalla, Saif Khan, Srushti Patel, Maria F Fleming, Princess T Oghomitse-Omene, Parth Patel, Muhammad Saqlain S Qavi

**Affiliations:** 1 Internal Medicine, Ghulam Muhammad Mahar Medical College Hospital, Sukkur, PAK; 2 Clinical Research, University of Tripoli, Tripoli, USA; 3 Medicine, California Institute of Behavioral Neurosciences and Psychology, Fairfield, USA; 4 Internal Medicine, Maharajah's Institute of Medical Sciences, Vizianagaram, IND; 5 Hospital Medicine, North Manchester Hospital, Manchester, GBR; 6 Pediatrics, Gujarat Medical Education and Research Society Medical College, Gandhinagar, IND; 7 Medicine, Forum of Artificial Intelligence in Medicine, Miami, USA; 8 Medicine, Universidad Central de Venezuela, Caracas, VEN; 9 Pediatrics and Neonatology, Delta State University Teaching Hospital, Abraka, NGA; 10 Pediatrics and Child Health, Covenant Community Care, Detroit, USA; 11 General Practice, Shri M. P. Shah Medical College, Jamnagar, IND; 12 Internal Medicine, Akhtar Saeed Medical and Dental College, Lahore, PAK

**Keywords:** uk - united kingdom, gdm- gestational diabetes mellitus, iq - intelligence quotient pdd- pervasive developmental disorders, opd- outpatient department, ent - ear nose and throat, lbw- low birth weight, spss- statistical package for the social sciences, or- odd's ratio, who- world health organization

## Abstract

Introduction

Communication is the exchange of information through speaking, writing, and other mediums. Speech is the expression of thoughts in spoken words. Language is the principal method that humans use for relaying information; consisting of words conveyed by speech, writing, or gestures. Language is the conceptual processing of communication. Problems in communication or oral motor function are called speech and language disorders. Developmental delay is diagnosed when a child does not attain normal developmental milestones at the expected age. Speech and/or language disorders are amongst the most common developmental difficulties in childhood. Such difficulties are termed 'primary' if they have no known etiology, and 'secondary' if they are caused by another condition such as hearing and neurological impairment, and developmental, behavioral, or emotional difficulties.

Objectives

The objective of our study was to observe the risk factors for speech and language delay in the children presenting to the speech therapy clinic of a tertiary care hospital in a large urban center.

Methodology

A cross-sectional study was conducted on 150 children presenting at the speech therapy clinic of Lahore General Hospital from July to August 2021. A well-designed questionnaire was used to collect data about the sociodemographic profile, and biological, developmental, and environmental risk factors of speech and language delay in children. SPSS, version 25 (IBM Corp., Armonk, NY) was used to enter and analyze the data.

Results

Parents or caretakers of a total of 98 male and 52 female children took part in this study aged 2-11 years. The average age of speech and language delay among the children was 5.65 years, 66.7% of which went to normal school while 31.3% went to special school; 66.7% were from urban areas. Around 60% had middle ear infections, and 34.7% were found to have oropharyngeal anomalies. A history of intrapartum complications was found in 68.4% of children; 46.7% of children had a history of use of a pacifier and 38% had a history of thumb sucking. Nearly 39% of children belonged to a multilingual family environment and 66.7% had a family history of screen viewing for more than two hours.

Conclusion

The major risk factors contributing to speech and language delay in children are family history of speech and language delay, prolonged sucking habits, male gender, oropharyngeal anomalies, hearing problems, and middle ear infections. Measures should be taken to educate people regarding risk factors, courses, and management of speech and language delay in children.

## Introduction

Speech is the most important form of conveying information and it can only be made possible through language. Language essentially embodies the words relayed via verbal or non-verbal ways. Disorders of speech and language could be defined as affliction in oro-motor function and dysfunction or lack of communication. Delayed speech and language can be identified when a patient does not achieve what is expected at an appropriate age [1].

Of the children going to primary schools, nearly 5% have a detected delay in speech and language. The global prevalence of these disorders in this age group varies between 3%-20% [2]. However, the percentage of speech and language disorders in school-going children is lower than the global average [3]. This could be one of the most significant hindrances to developmental difficulty in any child, which can be termed as primary if no possible etiology could be diagnosed. However, if a diagnosis is established, it can be referred to as a secondary cause. These causes can be classified as hearing difficulty, behavioral or emotional problems, and/or neurological causes [4].

As per the U.S. Preventive Services Task Force, the causative factors for these disorders consist of family history, premature birth, intrauterine growth retardation (IUGR) male gender, and parents of low socioeconomic background [5]. Nearly 4/5th of school-going children suffer from at least one episode of otitis media in their school life that can lead to delayed speech and language. Suckling in children has previously been linked to speech and language disorders. Excessive sucking of pacifiers, dummies, thumbs, and/or bottles can cause a decreased sense of the oral cavity and could also lead to oro-motor dysfunction. Family history of speech and language disorders has a strong association with a delay in speech and language [6]. Nearly half of the children with such disorders have a positive family history; the type of disorder, however, can vary [7].

All children must be screened for speech, language, and hearing difficulties. A delay in diagnosis and management can lead to a permanent loss in cognitive development leading to low intelligent quotient (IQ), difficulty in communication, and illiteracy [7]. There is a vast majority of evidence in the support of speech therapy in the setting of these disorders. Idiopathic etiologies have a better prognosis. An assessment of risk factors could lead to devising strategies in achieving the prevention of these disorders.

## Materials and methods

This was a cross-sectional study. The study was conducted in the speech therapy clinic at a tertiary care public hospital in a large urban center in Pakistan. The hospital is 1196 bedded teaching hospital and has 31 departments. There is only one speech therapy clinic on the second floor of the outpatient department (OPD) building. On average, the speech therapy clinic receives 2-3 patients per day. The study population included all children with speech and language delays who presented to the speech therapy clinic of Lahore General Hospital. The data were collected from July 2021 to August 2021. The approval (00/67/20) was obtained from the IRB of Lahore General Hospital before data collection.

The sample size was estimated using the World Health Organization (WHO) sample size software by using the formula of estimating population production with specified relative precision. With a confidence level of 95% and an anticipated population proportion of 73% with a relative precision of 10%, the minimum sample size was 150. The sampling technique used was nonprobability convenient sampling because of limited resources and a shortage of time. All children who presented to the speech therapy clinic of Lahore General Hospital were included. However, children diagnosed with autism spectrum disorder were excluded alongside those whose attendants refused after informed consent was provided.

Data were collected using a well-structured questionnaire after obtaining informed consent. The questionnaire consisted of four main parts. Sociodemographic profile, biological factors, family-based risk factors, and environmental factors. Data were collected with the help of face-to-face interviews with attendants of patients (children) coming to the speech therapy clinic of the Lahore General Hospital. A predesigned structured questionnaire was used. Data were collected by a group of eight doctors and students, and the questionnaire was translated into the local language for convenience. The questionnaires were checked for completeness every day.

SPSS software, version 25 (IBM Corp., Armonk, NY) was used for the entry, analysis, and computation of the data. For quantitative variables, the mean, median, and standard deviation were calculated. For qualitative variables, frequency distribution tables and percentages were generated. Data are presented using frequency tables, charts, and graphs. Descriptive analysis was used for sociodemographic and categorical data. The variables associated with speech and language delay were analyzed using bivariate analysis. A p-value of less than 0.05 and a confidence interval of 95% were considered statistically significant.

## Results

A cross-sectional study was conducted in July and August 2021 on children presenting to the speech therapy clinic of Lahore General Hospital. Data were collected from attendants of 150 children using a well-structured questionnaire by a nonprobability convenience sampling technique. From the sociodemographic profile of the patients, the following results were obtained. Table 1 describes the demographic profiling of the participants.

**Table 1:** Descriptive analysis of demographic factors

Variables	Categories	Frequency (N)	Percentage (%)
Gender	Male	98	65.3
	Female	52	34.7
Paternal education	Illiterate	26	17.3
	Primary/Middle school	50	33.3
	High school	46	30.7
	Graduate and higher	28	18.7
Maternal education	Illiterate	34	22.7
	Primary/Middle school	70	46.7
	High school	10	6.7
	Graduate and higher	36	24.0
Father's occupation	Unemployed	13	8.7
	Employed	137	91.3
Nature of occupation	Job	75	50.0
	Businessman	75	50.0
Mother's occupation	Housewife	104	69.3
	Working	46	30.7
Place of residence	Rural	50	33.3
	Urban	100	66.7
Socio-economic status	Upper	11	7.3
	Middle	91	60.7
	Lower	48	32.0
Type of school child goes to	Normal	103	68.7
	Special	47	31.3
No of siblings	One	15	10.0
	Two	51	34.0
	Three	32	21.3
	Four	32	21.3
	Five	9	6.0
	Six	7	4.7
	Seven	1	7
	Eight	3	2.0
Child's birth order	First	67	44.7
	Second	45	30.0
	Third	21	14.0
	Greater	17	17.3

The respondents were group matched for age. The mean age of the respondents was 5.65 years, and the standard deviation was ±2.66 years. Almost half (68) of the total 150 patients had a significant injury, illness, or hospitalization. Of the total sample, 114 also had hearing problems. Table 2 describes the frequency of probable causes for speech and language disorders.

**Table 2:** Frequency of biological factors

Variables	Categories	Frequency (N)	Percentage (%)
Any significant injury, illness, or hospitalization	Yes	68	45.3
	No	82	54.7
Any seizure disorder	Yes	58	38.7
	No	92	61.3
Any hearing problem	Yes	114	76
	No	36	24.0
Ever had a middle ear infection	Yes	89	59.3
	No	61	40.7
Any other illness related to ear, nose, and throat (ENT)	Yes	74	49.3
	No	76	50.7
Consanguinity of parents	Yes	63	42.0
	No	87	58.0
Any oropharyngeal deformity	Yes	52	34.7
	No	98	65.3
Age of father at child's birth	Greater than 40 years	62	41.3
	Less than 40 years	88	58.7
Age of mother at child's birth	Greater than 40 years	64	42.7
	Less than 40 years	86	57.3

We found that 40.0% and 34.7% of the mothers had a history of hypertensive disorder during pregnancy and gestational diabetes, respectively. A history of anemia during pregnancy was present in 34.0% of the mothers. A history of fetal distress was found in 22 of the patients. The children with a history of neonatal seizures, prematurity, and low birth weight were 27.3%, 28.0%, and 33.3%, respectively.

Breastfeeding history was present in 78 children, while 72 children were bottle feeders. A history of thumb sucking was positive in 38.0% of all children. A history of pacifier use was found in 46.7% of the children (Table 3).

**Table 3:** Frequency table of feeding/developmental risk factors

Variables	Categories	Frequency (N)	Percentage (%)
Feeding history	Breastfeeding	78	52.0
	Bottle-fed	92	48.0
History of thumb sucking	Yes	57	38.0
	No	93	62.0
History of use of pacifiers	Yes	70	46.7
	No	80	53.3

Most of the children belonged to the joint family system (64.0%), while the rest were from the nuclear family (36%). No family members greater than four were present in 35.3% of the total cases. A family history of speech and language disorder was found to be positive in 98 of the total presented cases. A total of 38.7% of the children lived in a multilingual family environment (Table 4).

**Table 4:** Frequency table of family-based risk factors for speech and language delay

Variables	Categories	Frequency (N)	Percentage (%)
Type of family	Joint	96	64.0
	Nuclear	54	36.0
No. of family members	Greater than four	53	35.3
	Equal to or less than 4	97	64.7
Family history of speech and language disorder	Present	98	65.3
	Absent	52	34.7
Mother-child separation	Yes	61	40.7
	No	89	59.3
Father’s absence from home	Yes	48	32.0
	No	102	68.0
Multilingual family environment	Yes	58	38.7
	No	92	61.3

A history of recent trauma or stress was detected in 28 children alongside other findings (Table 5).

**Table 5:** Frequency table of environmental factors for speech and language delay

Variables	Categories	Frequency (N)	Percentage (%)
History of recent trauma or stress	Yes	28	18.7
	No	122	81.3
Screen Viewing (television, mobile, or laptop)	Greater than two hours	100	66.7
	Equal to or less than two hours	50	33.3

Various risk factors were compared with each other to determine which were significant. A chi-square test was applied. The associations analyzed are provided below in Tables 6-9.

**Table 6:** Association between gender of child and family history of speech disorder

Family history of speech and language disorder			Chi-square P-value
	Present (n)	Absent (n)	
Gender of child			0.073
Male	69	29	
Female	29	23	

**Table 7:** Association between a history of maternal hypertension and oropharyngeal deformity

History of hypertensive disorder pregnancy			Chi-square P-value
	Yes n (%)	No n (%)	
Any oropharyngeal deformity			0.141
Yes	25 (41.7	27 (30.0)	
No	35 (58.3)	63 (70.0)	

**Table 8:** Association between screen time and family history of speech disorder

Any hearing problem			Chi-square
	Yes (n)	No (n)	P-value
Screen viewing			0.043
Greater than two hours	71	43	
Equal to or less than two hours	29	7	

**Table 9:** Association between consanguinity of parents and family history of speech disorder

Consanguinity of parents			Chi-square
	Yes n (%)	No n (%)	P-value
Family history of speech and language disorder			0.182
Present	45 (45.9)	18 (34.6)	
Absent	53 (54.1)	34 (65.4)	

Results indicate there are risk factors for developing a speech and language disorder with being a male, being born as the first child, being born in a joint family and parents who are illiterate, and those who have a family history of disorders. A positive association has been established, as shown in the tables above (Tables 6-9).

## Discussion

When a child's speech is incomprehensible or does not achieve what is required at a specific age, it can be referred to as a speech and language delay. Major risk factors can be divided into antenatal, neonatal, or developmental. Maternal participation that is widely concerned with developing communication in children includes motivating the child to speak, imparting elaborative remarks, storytelling, and involving the child in reading [7]. The literacy of fathers and mothers also affects the development and speech of a child. Among the parents who came with their children with delayed speech and language, 22.7% of mothers were illiterate, 46.7% had primary education, 6.7% had higher secondary education, and 24% were graduates. Mondal et al. also indicated that maternal illiteracy is a risk factor for speech and language delay [8].

Psychological disorders in parents, breastfeeding, the interaction of siblings, and the size of the family have a significant impact on the development of speech and language [7]. Our study showed that the average age of children with speech and language delay was 5.5 years, ranging from 2-11 years of age. Out of 150 respondents, 98 (65.3%) were male, and 52 (35.7%) were female, indicating that males are at a higher risk for developing speech and language delays. Similar results were shown in a study by Mondal et al. where 33% of male children and 19% of female children presented with speech and language delay [8].

Barry et al. found that all parents of affected children had a family history of language or speech disorders. Around 24% also had a first-degree relative with the disorder [9]. Our study showed that approximately 65.3% of children had a previous family history of speech and language delay, indicating that family history is a major risk factor for speech and language delay, similar is the case with maternal hypertension during pregnancy [8,10]. Yasin et al. observed that 23% of the patients presenting with speech disorders or delays also have a psychiatric diagnosis and it is important to evaluate these patients with a multidisciplinary team and refer them to the mental health clinic for the screening of psychological disorders [11].

A positive association between language delay and frequency of screen time was observed. Children who developed delayed speech and language began watching television (TV) at the age of 7 ± 5 months vs. 12 ± 5 months in normal children and consumed increased time watching TV i.e. 3 ± 1.90 h/day vs. 1.85 ± 1.18 h/day in normal children. Children who began watching TV at <12 months of age and watched TV for more than two hours a day were nearly at a six times higher risk of developing delayed language and speech [12].

Tan et al. concluded that a supportive environment at home with absolute breastfeeding and a harmonious family environment in the initial years of development considerably helped in attaining language skills [13]. Multivariate analysis revealed that exclusive breastfeeding for <6 months, delayed gross motor milestones, >2 hours/day of screen time, and deficient social exchange are significant risk factors for delayed speech in children [14]. According to the National Committee on Vital and Health Statistics at the Department of Health and Human Services, Washington, nine factors were constantly recognized as having a distinctive impact on delayed language and speech. Risk factors included male sex, the presence of hearing disorders, and impulsive behavior. While protective factors included having a more persistent nature, being socially active, and good maternal health. Lastly, the factors that could be either risky or protective comprised having an elder sibling, parental LOTE (languages other than English), and a supportive learning environment at home [15].

Our results showed that 44.7% of the children who presented with a delay in speech and language were first born, 30% were second born, and the remaining children were of greater order, which indicates that the prevalence of speech and language delay is higher in first-born children of the family, which is due to the lack of experience of parents regarding the child’s development. However, the study by Mondal et al. shows that higher prevalence is found in those of the third or greater order [11]. This study shows that children with prolonged sucking habits are more prone to speech and language delays. Fox et al. stated that sucking habits were a significant factor in speech and language delay [10].

Most of the children with speech and language delays were living in the joint family system (64%), whereas the remaining 36% were living in the nuclear family system, which shows that the prevalence of speech and language delays is high among children living with joint/large families. Many studies favor this factor as a risk factor for speech and language delay, except the study by Fox et al. in the UK which observed that unilingual families pose a higher risk of speech and language delay [8,10].

Of the children included in our study, 27% had a history of neonatal seizures, 72% had a preterm birth, and 33.3% had a low birth weight. Similar results were shown in another study in which 31% of children with speech and language delay were born with low birth weight and 14% were born preterm [16]. In our study, 59.3% had a history of middle ear infection, 34.7% were found to have oropharyngeal disorders, and 76% had associated hearing problems. Similar findings were observed in studies conducted in various settings [2,8,10] 

Chonchaiya et al. suggested that speech and language therapy is effective for children with phonological or vocabulary difficulties. No remarkable difference was observed between clinician-administered therapy and that implemented by trained parents [16]. Speech and language delay causes impairment of intelligence of the child and development of mental capabilities; therefore, timely diagnosis is necessary to prevent the long-term effects of delay. Children who are exposed to any risk factor for speech and language delay should be monitored and taken to the speech therapy clinic for a checkup. However, any underlying cause of the delay should be screened and treated first.

Limitations

This was a hospital-based cross-sectional study, and only patients arriving at one hospital were included. Since the study addresses some personal and sensitive behavior, there is a possibility of falsified reporting among attendants of children, especially given the face-to-face interview modality of data collection. Other limitations include potentially uncontrolled confounding effects and reporting bias due to the self-reported nature of the data collection method.

## Conclusions

From the study, it was found that factors that contributed the most to the speech and language delay in children were male gender, long-term sucking habits, illiteracy of the mother, preterm birth, low birth weight, oropharyngeal deformity, hearing problems, intrapartum or postpartum complications and previous family history of speech and language delay. The less significant factors were low socioeconomic status, order of the child, occupation of father and mother, socioeconomic status, and no family members.

Parents should be educated regarding the effects of speech and language delay on their children and how to avoid preventable risk factors. Special care should be given to females during pregnancy and the postpartum period. Children should be monitored carefully for delay of milestones, especially regarding speech, and care should be sought if a delay is observed. Speech therapy is recommended in any case of speech and language delay for proper diagnosis and treatment.
